# Analysis of determinants for *in vitro* resistance to the small molecule deubiquitinase inhibitor b-AP15

**DOI:** 10.1371/journal.pone.0223807

**Published:** 2019-10-22

**Authors:** Arjan Mofers, Paola Perego, Karthik Selvaraju, Laura Gatti, Joachim Gullbo, Stig Linder, Padraig D'Arcy

**Affiliations:** 1 Department of Medicine and Health, Linköping University, Linköping, Sweden; 2 Molecular Pharmacology Unit, Department of Applied Research and Technological Development, Fondazione IRCCS Istituto Nazionale dei Tumori, Milan, Italy; 3 Cerebrovascular Unit, Department of Clinical Neurosciences, Fondazione IRCCS Istituto Neurologico Carlo Besta, Milan, Italy; 4 Department of Radiology, Oncology and Radiation Science, Section of Oncology, Uppsala University, Uppsala, Sweden; 5 Department of Oncology-Pathology, Karolinska Institutet, Stockholm, Sweden; Wayne State University, UNITED STATES

## Abstract

**Background:**

b-AP15/VLX1570 are small molecule inhibitors of the ubiquitin specific peptidase 14 (USP14) and ubiquitin carboxyl-terminal hydrolase 5 (UCHL5) deubiquitinases (DUBs) of the 19S proteasome. b-AP15/VLX1570 have been shown to be cytotoxic to cells resistant to bortezomib, raising the possibility that this class of drugs can be used as a second-line therapy for treatment-resistant multiple myeloma. Limited information is available with regard to potential resistance mechanisms to b-AP15/VLX1570.

**Results:**

We found that b-AP15-induced cell death is cell-cycle dependent and that non-cycling tumor cells may evade b-AP15-induced cell death. Such non-cycling cells may re-enter the proliferative state to form colonies of drug-sensitive cells. Long-term selection of cells with b-AP15 resulted in limited drug resistance (~2-fold) that could be reversed by buthionine sulphoximine, implying altered glutathione (GSH) metabolism as a resistance mechanism. In contrast, drug uptake and overexpression of drug efflux transporters were found not to be associated with b-AP15 resistance.

**Conclusions:**

The proteasome DUB inhibitors b-AP15/VLX1570 are cell cycle-active. The slow and incomplete development of resistance towards these compounds is an attractive feature in view of future clinical use.

## Background

Cancer cells display high rates of protein synthesis and depend on the ubiquitin-proteasome system (UPS) for maintenance of homeostasis. Cancers with high rates of protein turnover are generally sensitive to proteasome inhibition, as exemplified by multiple myeloma cells that express elevated levels of immunoglobulin chains. Such cells experience a continuous state of proteotoxic stress due to their predisposition to accumulate defective proteins. The clinical development and subsequent approval of the 20S proteasome inhibitor bortezomib for multiple myeloma validated the UPS as a therapeutic target [1]. Bortezomib is a peptide boronate that inhibits the chymotrypsin-like activity of the β5-subunit and the caspase-like activity of the β1-subunit of the 20S proteasome. The success of bortezomib has led to large efforts in identifying additional proteasome inhibitors with different mechanisms of action [2]. The epoxyketone carfilzomib targets the chymotrypsin activity of the 20S proteasome [3]. A major difference between bortezomib and carfilzomib is the extent of drug occupancy, carfilzomib being an irreversible inhibitor and bortezomib displaying a slowly reversible inhibitory mechanism. Ixazomib (MLN9708), the first oral proteasome inhibitor approved by FDA, preferentially inhibits the chymotrypsin-like activity of the β5 subunit similarly to bortezomib.

The majority of patients treated with bortezomib eventually acquire resistance and relapse. A number of molecular mechanisms underlying bortezomib resistance have been described (for recent reviews, see [4, 5]). Mutations in the genes encoding the catalytic β-subunits have been observed in cell lines selected for bortezomib resistance *in vitro* [6–10]. The clinical picture is less clear since mutations in β5 are generally absent from clinical specimens displaying bortezomib-resistance [5]. ABC-drug efflux pump activity has been implicated in bortezomib resistance although the evidence is not altogether clear [11–14]. Furthermore, overexpression of members of the Bcl-2 family of anti-apoptotic mediators has been associated with bortezomib resistance [15, 16] and it has been shown that the anti-tumour activity of bortezomib is enhanced by Bcl-2 antagonists [17–19]. It was recently demonstrated that bortezomib resistance in multiple myeloma cell lines and patient cells is associated with increased serine synthesis [20].

The small molecule b-AP15 [(3E,5E)-3,5-bis[(4-nitrophenyl)methylidene]-1-(prop-2-enoyl)piperidin-4-one] and its derivative VLX1570 have been described as potent inhibitors of proteasome deubiquitinases. Deubiquitinase activity is required for proper proteasome function due to the necessity to remove ubiquitin from misfolded protein substrates prior to proteolytic processing. The proteasome contains two cysteine deubiquitinases (USP14 and UCHL5) and one metalloprotease (Rpn11/POH1) that perform this task. b-AP15/VLX1570, as well as other chemically related compounds, display promising therapeutic activities in a number of tumor models, including multiple myeloma [21, 22], Ewing’s carcinoma [23] Waldenströms macroglobulinaemia [24], mantle cell lymphoma [25], hepatocellular carcinoma [26], prostate cancer [27], breast cancer [28], melanoma [29], colon cancer [30], neuroblastoma [31] and lung carcinoma [32]. The utility of these compounds is limited by low solubility, resulting in the use of Kolliphor EL for administration and restricting the clinical use of VLX1570 (clinical trial NCT02372240 currently on hold). PEGylation may provide a resolution to this problem [33] and a Kolliphor-free formulation of b-AP15 has recently been described [29].

The presence of α,β-unsaturated ketones (enones) in b-AP15 is believed to target the nucleophilic cysteines in the active sites of USP14 and UCHL5. b-AP15 has been shown to be cytotoxic in cells resistant to bortezomib [21, 34], but limited information is available with regard to potential resistance mechanisms to b-AP15. We have here studied this problem and report that only modest resistance to b-AP15 develops *in vitro* despite long-term continuous exposure.

## Results

### Selection of cells for resistance to b-AP15

**Fig 1:**
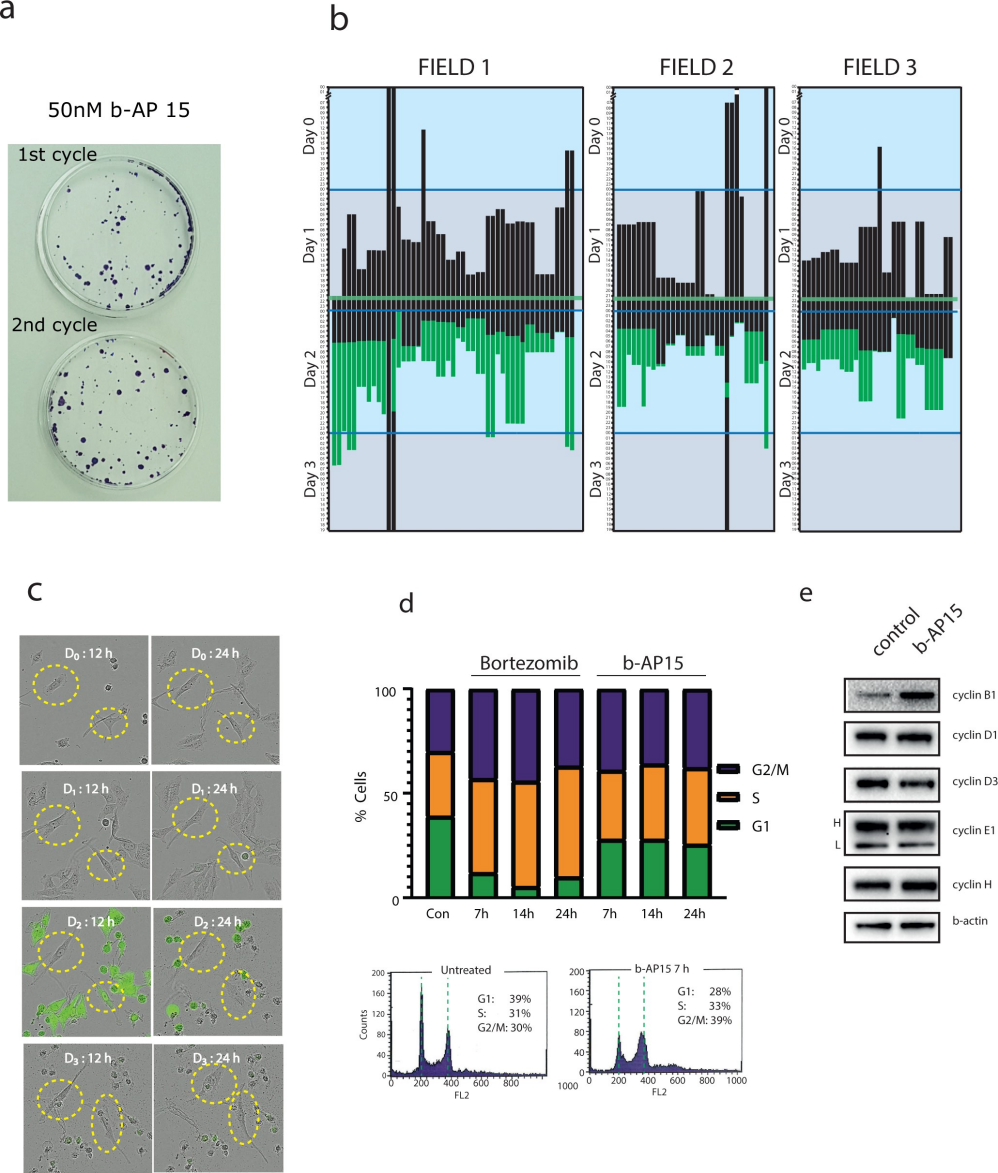
Effect of b-AP15 on tumor cell proliferation. **a.** Clonogenic assay for HCT116 cells exposed to 50 nM b-AP15 for one or two cycles. There is no clear increase in resistance to b-AP15 on the second treatment cycle compared to the first, indicating cells surviving the first treatment cycle did not acquire resistance to b-AP15. Similar results were observed in consecutive cycles. **b.** Lineage tracing of MelJuSo Ub-YFP cells by live cell imaging. The starting point of each black bar indicates a cell division. The bar turning green indicates visual accumulation of Ub-YFP, indicating proteasome inhibition. The bar ending indicates death of the cell. Drug was added on day 1, indicated by the horizontal green line. **c.** Live cell images of lineage tracing experiment, with two cells that survived indicated treatment. Accumulation of Ub-YFP can be observed as yellow/green coloured cells. **d.** Cell cycle analysis of HCT116 cells exposed to bortezomib or b-AP15. Above: Stacked column graph showing distribution between phases of the cell cycle for cycling cells after treatment with bortezomib (100 nM) or b-AP15 (1 μM) for indicated times. Data based on cellular DNA content (PI staining) analyzed by flow cytometry. Below: Single-parameter histogram showing DNA content (PI staining) in response to 7h b-AP15 treatment (1 μM) compared to control. Representative histogram for data visualized in the stacked column graph shown above. **e**. Analysis of cyclin levels in cells exposed to b-AP15 for 6 hours using immunoblotting. H and L: high and low molecular weight forms of cyclin E; the L molecular form is often observed in tumors [59].

We previously reported that attempts to derive b-AP15 resistant multiple myeloma cell lines by selection were unsuccessful [34]. We here made a new attempt of selection using the b-AP15-sensitive colon cancer cell line HCT116. These cells are known to be sensitive to b-AP15 (see NCI profile below) and, in contrast to myeloma cells, are adherent, facilitating outgrowth of clones of resistant cells. Cells were exposed to low concentrations of b-AP15, resulting in massive cell death (> 99%) and outgrowth of colonies (Fig 1A). Surviving colonies were pooled and re-exposed to b-AP15 at the same concentration as initially used. Surprisingly, the same effect of massive cell death and clonal outgrowth was observed in the second cycle of treatment (Fig 1A). The procedure was repeated using the same or slightly higher drug concentrations. Similar results were obtained, i.e. the outgrowth of colonies that were found to show limited or no resistance to the drug. After > 6 months of repeated selection steps, a HCT116 derivative was obtained that showed ~2-fold resistance to b-AP15 (HCT116^b-AP15^). These cells showed the same level of resistance to the structurally related analogue VLX1570. The same selection procedure was performed in parallel using bortezomib and resulted in the isolation of cells showing >10-fold resistance (IC_50_ > 800 nM compared to 60 nM of parental cells).

### Sensitivity to b-AP15 is associated with active cell proliferation

The survival of cells that did not show resistance suggested a stochastic phenomenon of drug killing. Such phenomena have been discussed and modelled [35] but not extensively studied. We hypothesized that evasion is related to differences in drug sensitivity during different phases of the cell cycle. To test this hypothesis, we performed time-lapse imaging experiments using a melanoma cell line expressing a proteasome degradable reporter substrate (MelJuSo Ub^G76V^ YFP [36]). The cells were followed for 46 h prior to drug exposure and for 46 h after exposure to 250 nM b-AP15 (Fig 1B).

The median time of cell death following drug treatment was 12 hours, although large variations were observed (from 5 hours to > 24 hours). A clear pattern with simultaneous cell death of daughter cells was observed (Fig 1B), showing that the variations in the lengths of the cell death process are non-random. No association between post-mitotic time at drug addition and the subsequent interval to cell death was observed (Figs 1B and S1). A small number (3/112) of tracked cells did not die during the observation period. Strikingly, none of these cells had undergone mitosis during the 46 hour period prior to drug addition (Fig 1B and 1C). Two of these cells were found to become weakly and transiently YFP-positive (Fig 1C), suggesting a phase of proteasome inhibition that was subsequently overcome. These experiments suggest that non-cycling HCT116 cells are relatively insensitive to b-AP15 and may re-enter the cell cycle to form surviving colonies.

Analysis of time-lapse movies showed that no mitotic events occurred after addition of b-AP15 (Fig 1B). The 20S proteasome inhibitor Bortezomib has previously been shown to induce G2/M arrest [37]. The cell cycle distributions of cells exposed to b-AP15 or the 20S proteasome inhibitor bortezomib were examined and found to be similar, but not identical. Both drugs were found to arrest cells in G2/M (Fig 1D). However, whereas a distinct G1 population was observed in cells exposed to b-AP15, this population was gradually lost in bortezomib-treated cells and an increasing number of cells in S-phase were observed (Fig 1D). Consistent with induction of G2/M arrest, b-AP15 treated cells were found to express increased levels of cyclin B1 (Fig 1E).

### 20S proteasome and general DUB activity

**Fig 2:**
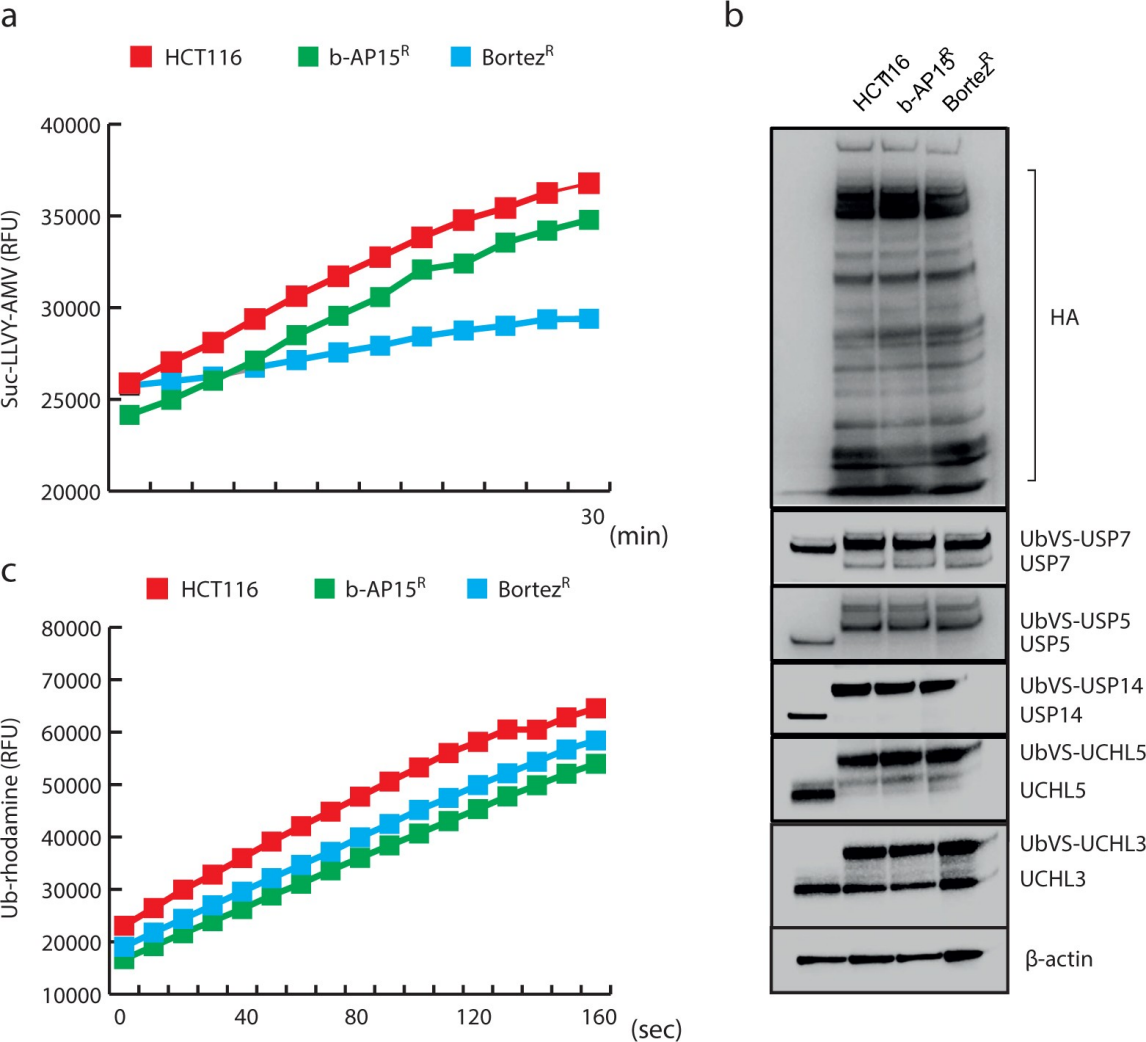
Proteasome activity in b-AP15- and bortezomib resistant cells. **a.** 20S proteasome activity of cell extracts prepared from parental, b-AP15-resistant and bortezomib-resistant HCT116 cells measured using Suc-LLVY-AMC substrate. b-AP15 resistant cells did not show altered 20S activity compared to parental type cells, whereas lower activity was observed in the bortezomib-resistant cell line. **b.** DUB activity of parental, b-AP15- and bortezomib-resistant HCT116 cells measured using active site probe UbVs-HA. DUB activity is visualized by HA antibody labeling. There is no observable difference in the activity of any of the DUBs analyzed in b-AP15-resistant or bortezomib-resistant cells compared to parental cells. **c.** Total DUB activity of cell extracts prepared from parental, b-AP15-resistant and bortezomib-resistant HCT116 cells measured using Ub-rhodamine substrate. No significant change in total DUB activity is observed in the resistant cell lines compared to the parental line.

To assess whether changes intrinsic to the target of b-AP15, the ubiquitin proteasome system, could lead to resistance to the drug, we analyzed whether the ubiquitin proteasome system showed any changes in activity in cells resistant to b-AP15 or bortezomib. No significant difference in the levels of 20S proteasome activity was observed between parental and HCT116^b-AP15^ cells, whereas bortezomib-resistant cells showed a decrease (Fig 2A). Furthermore, total DUB activity and the activity of USP14, UCHL5 and other DUBs visualized to UB-VS labeling were not affected (Fig 2B).

### b-AP15-resistance is glutathione dependent

**Fig 3:**
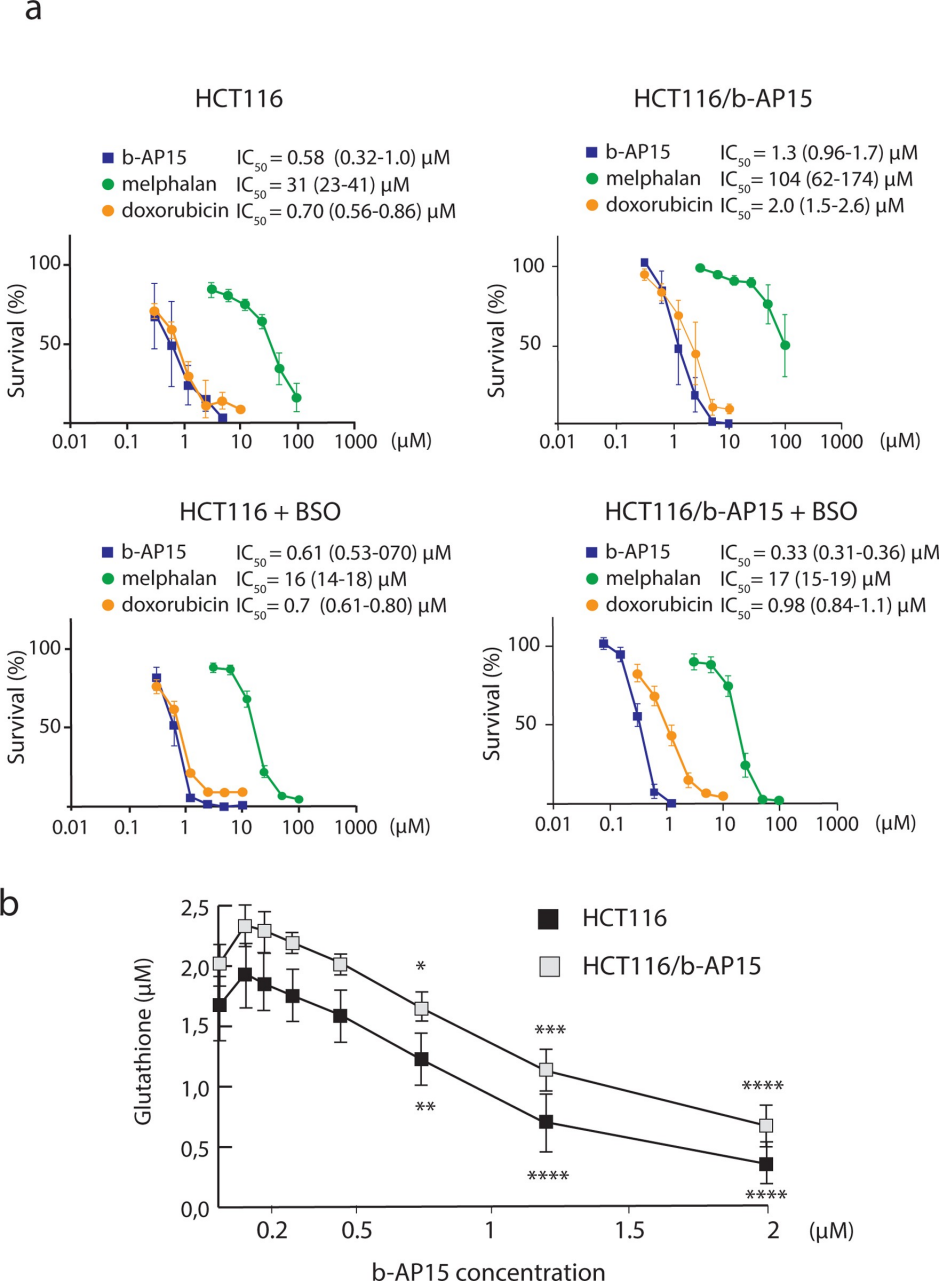
Comparison of IC_50_ for anti-cancer drugs in parental versus b-AP15 resistant HCT116 cells and effect of glutathione depletion. **a.** b-AP15-resistant HCT116 cells show similar and modest resistance to b-AP15 (2.2-fold), melphalan (3.4-fold) and doxorubicin (2.9-fold) compared to parental HCT116 cells. The sensitivity to b-AP15, melphalan and doxorubicin is increased by glutathione depletion of the b-AP15 selected cell line. Indicated are IC50 values ± 95% confidence interval. **b.** b-AP15-resistant HCT116 cells show a significant increase in GSH levels at baseline (P = 0.019, Student’s T-Test, 2-tailed, unequal variance assumed). GSH levels are increased in response to low concentrations of b-AP15, whereas high concentrations of b-AP15 cause significant depletion in GSH. * = P < 0.05. ** = P < 0.005. *** = P < 0.0005. **** = P < 5 x 10^−5^. (Student’s T-Test, 2-tailed, unequal variance assumed).

We examined the sensitivity of HCT116^b-AP15^ cells to other anticancer drugs. The cells showed a ~3-fold increased IC_50_ to the alkylating agent melphalan and to doxorubicin (Fig 3A). Although the sensitivity of both these drugs is known to be dependent on multiple factors, GSH metabolism is a common feature [38, 39]. To test the possible influence of GSH on b-AP15 sensitivity we used buthionine sulphoximine (BSO) to deplete GSH. BSO is a commonly used inhibitor of γ-glutamylcysteine synthetase, a key enzyme in GSH biosynthesis [40]. We found that the sensitivity of HCT116^b-AP15^ cells to b-AP15 was increased by BSO pre-treatment, as was the sensitivity to melphalan and doxorubicin (Fig 3A). In contrast, the sensitivity of parental HCT116 cells to b-AP15 was not detectably affected by BSO treatment.

We further assessed GSH levels in wildtype and resistant cells and the effect of b-AP15 on GSH levels in these cells. GSH levels were significantly elevated in HCT116^b-AP15^ cells compared to HCT116^WT^ (2.14μM vs 2.02μM GSH, P = 0.019, Fig 3B). Concentrations of b-AP15 up to ~0.3μM cause elevated GSH levels compared to controls indicating a stress response, whereas significant depletion of GSH was observed for higher concentrations of b-AP15. HCT116^b-AP15^ cells maintained elevated levels of GSH at all probed concentrations of b-AP15 compared to HCT116^wt^, indicating less severe depletion of GSH even at high concentrations of b-AP15 in resistant cells.

### b-AP15 resistance is not associated with expression of drug transporters

**Fig 4:**
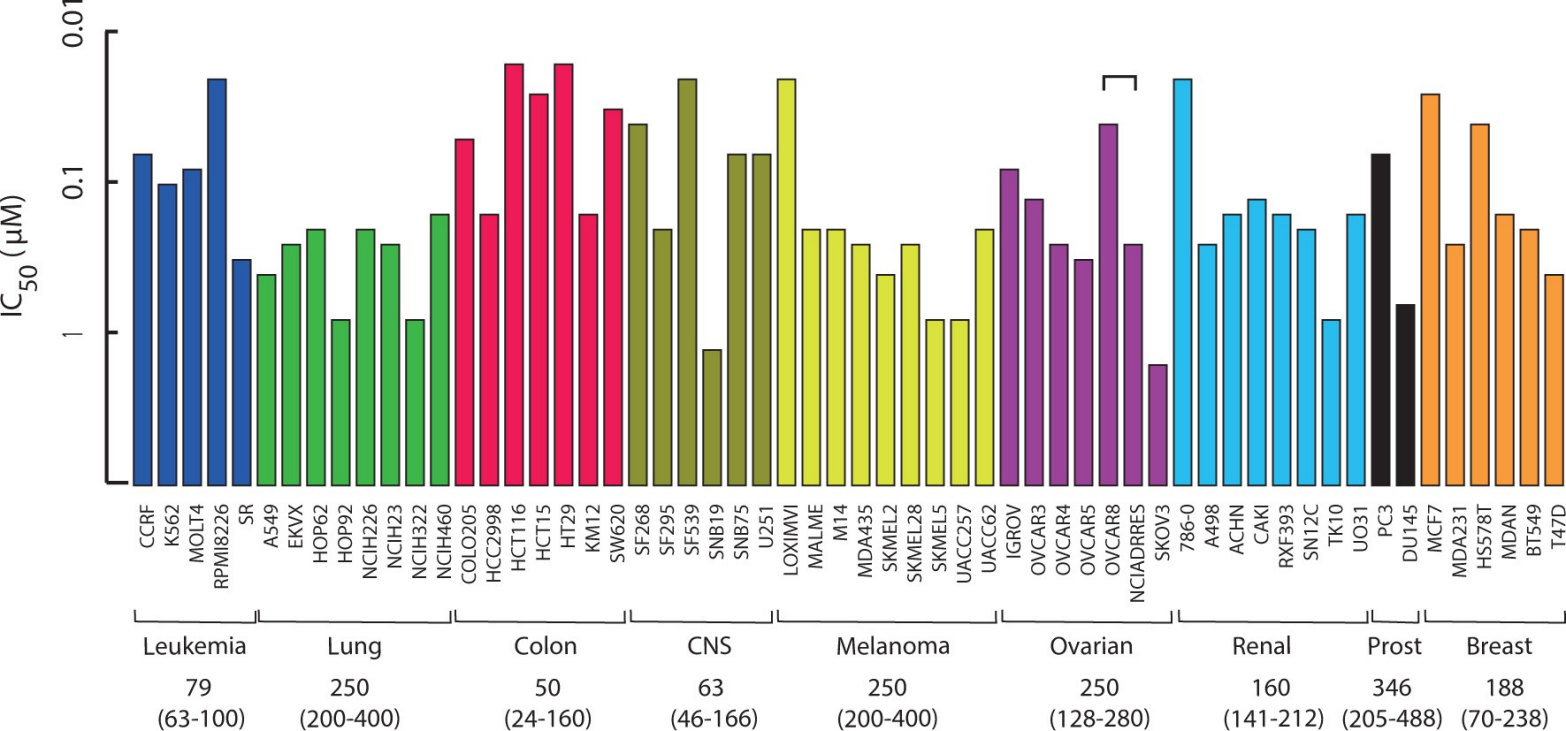
Cell sensitivity of the NCI_60_ cell line panel to b-AP15. Data retrieved from NCI Developmental Therapeutics Program database (https://dtp.cancer.gov/), *in vivo* screen summary data.

In addition to changes in GSH metabolism, overexpression of drug efflux pumps and alterations in topoisomerases have been implicated in doxorubicin resistance [41]. We examined the cell sensitivity profile of cells in the NCI_60_ cell line panel to b-AP15 (NSC687852) (https://dtp.cancer.gov/, 2016 dataset). Colon and CNS cancer cells were most sensitive to b-AP15 in the panel, whereas melanoma, prostate and lung cancers showed the least sensitivity (Fig 4). Interestingly, the adriamycin/doxorubicin-resistant variant of OVCAR-8 cells (NCI/ADR-RES) were ~7-fold less sensitive to b-AP15 compared to parental OVCAR-8 cells (Fig 4). The finding of cross-resistance adriamycin/doxorubicin is consistent with the findings from the HCT116^b-AP15^ cells. It should be pointed out, however, that the level of resistance to b-AP15 is modest compared to the > 100-fold resistance displayed by NCI/ADR-RES cells to doxorubicin [42].

The expression levels of 48 different ABC transporters have been determined in the NCI_60_ cell line panel [43], and we calculated the Spearman correlation coefficients between b-AP15 IC_50_ values in the NCI_60_ and the expression levels of those transporters. No statistically significant correlations were observed after Bonferroni adjustment (S1 Table). Drug sensitivity is also influenced by the extent of transporter-mediated drug uptake. The solute carrier (SLC) family consists of a number of transporter and exchange proteins that facilitate substrate transport, including that of anticancer drugs such as methotrexate (SLCO1B3) [44] and platinum compounds (SLC22A1-3) [45]. We examined the possible association between solute carrier expression (SLC22 family and SLCO family) and sensitivity to b-AP15 using the expression data of [46]. No statistically significant correlations between expression levels and sensitivity were observed (S2 Table).

Overexpression of the ABC transporter MDR1 has been demonstrated in NCI/ADR-RES [47]. We directly tested a series of cell lines that over-express ABCB1 (MDR1/P-gp), ABCC1 (MRP1), ABCC4 (MRP4) or ABCG2 following transfection of full-length cDNA. Increased expression of any of these transporters, including MDR1, did not result in decreased sensitivity to b-AP15 (Table 1). Expression of ABCC1 (MRP1) did, on the contrary, result in 2.8-fold increased sensitivity to b-AP15.

**Table 1:** Sensitivity of cells over-expressing different transporters of the ABC superfamily to b-AP15.

Cell line	IC_50_ (nM) (mean ± S.D.)
U2-OS	89 ± 12
U2-OS empty vector	120 ± 30
U2-OS/MRP1^a^	32 ± 10
U2-OS/MRP4^b^	140 ± 20
HT29	210 ± 30
HT29/MIT^c^	220 ± 90
LoVo	140 ± 10
LoVo/DX^d^	130 ± 6
A2780	49 ± 10
A2780/BBR	72 ± 30

Cell sensitivity was assessed by growth-inhibition assay. Cells were exposed to drugs for 72 h and counted at the end of treatment. IC_50_ represents the drug concentration producing 50% decrease of cell growth.

^a^ Cells characterized by over-expression of MRP1/ABCC1 following transfection of full-length cDNA.

^b^ Cells characterized by over-expression of MRP4/ABCC4 following transfection of full length cDNA.

cCells stably over-expressing ABCG2 (BCRP) following selection wit mitoxantrone.

^d^Cells stably over-expressing ABCB1 (P-gp/GP170) following selection with doxorubicin.

We also tested the sensitivity to b-AP15 of different cell lines selected for drug resistance. Doxorubicin resistance of LoVo/DX cells has been associated with overexpression of MDR1/PgP [48]. These cells were not, however, resistant to b-AP15 (Table 1). HT29/Mit cells, selected for resistance to mitoxantrone and overexpressing BCRP (breast carcinoma resistance protein) [49], did not display b-AP15 resistance (Table 1). Finally, the platinum-resistant cell line A2780/BBR, characterized by increased GSH metabolism compared to the parental cell line A2780 [50] showed a slightly reduced sensitivity to b-AP15 (Table 1).

### Measurements of drug uptake

**Fig 5:**
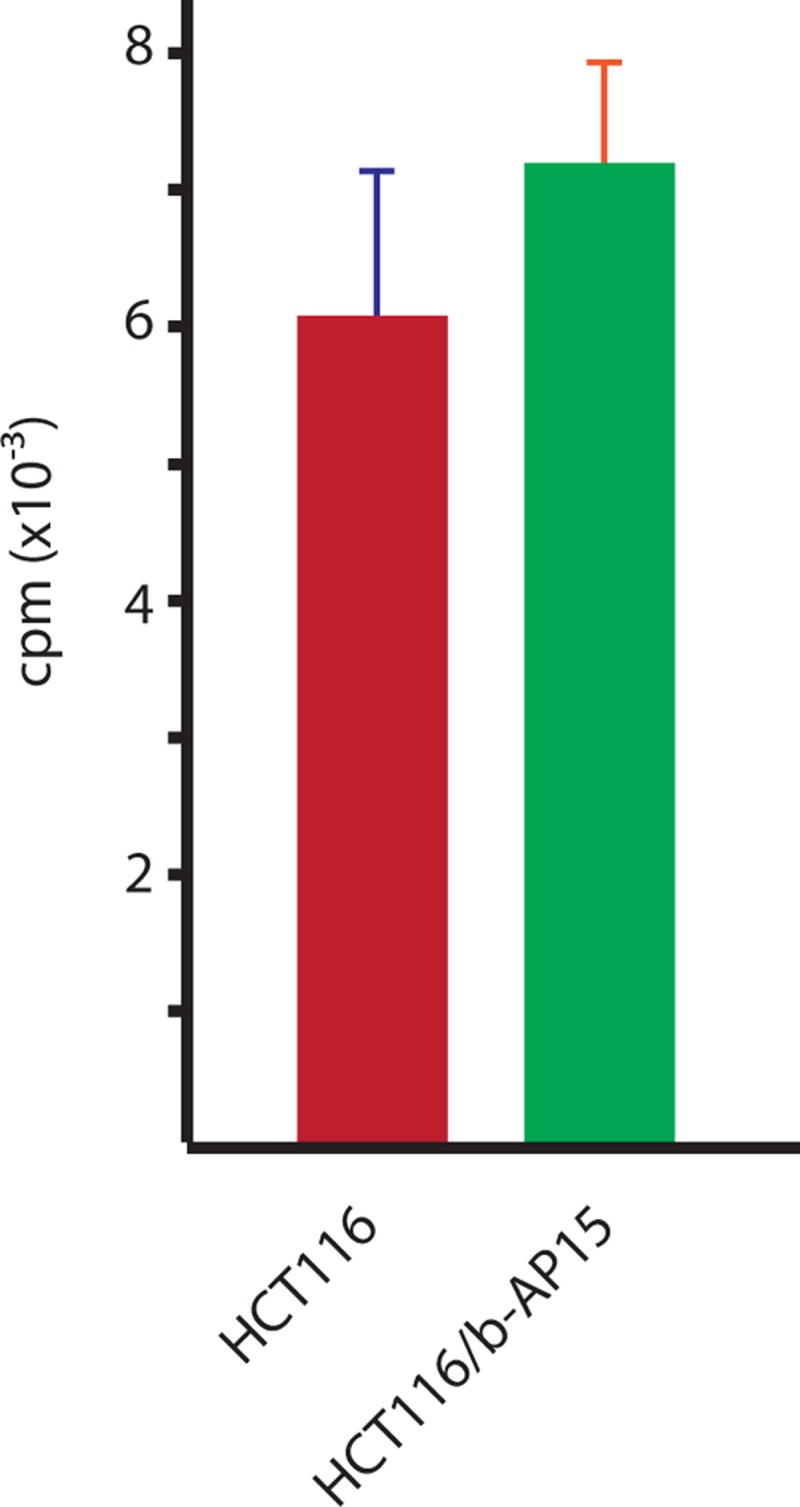
Uptake of ^3^H-labelled VLX1570 in parental and b-AP15-selected HCT116 cells. Cells were incubated with ^3^H-labelled VLX1570 and washed. Compound remaining in the cells was detected using a scintillation counter. There is no significant difference in uptake between parental and b-AP15-selected cells (Student’s T-Test, two-tailed, unequal variance assumed). VLX1570 is an optimized lead of b-AP15 showing the same mechanism of action [51] and b-AP15-resistant HCT116 cells are cross-resistant to VLX1570.

We directly tested whether drug uptake differed between parental and a HCT116^b-AP15^ cells. We only had access to ^3^H-labelled VLX1570, an optimized analogue of b-AP15 [34, 51]. Both b-AP15 and VLX1570 have been shown to be rapidly taken up by cells *in vitro* [34, 52] and b-AP15-resistant cells were cross-resistant to VLX1570. There was no significant difference in VLX1570 uptake between parental and resistant cells (Fig 5).

## Discussion

The results of the present and a previous study [34] show that resistance to the DUB inhibitors b-AP15/VLX1570 develop slowly and incompletely. We initially interpreted this phenomenon to mutations of the DUB target enzymes leading to loss of enzymatic activity and to loss of proteasome function. This would be in distinction to mutations in the β5-subunit of the 20S proteasome that will affect bortezomib binding with maintained proteasome function [5]. Further studies showed that non-cycling HCT116 cells were insensitive to b-AP15 and the results suggest that such non-cycling cells can re-enter the cell cycle and form new colonies with the same clonal characteristics as the original cells. This phenomenon of evasion of drug-induced cell death is likely to contribute to the slow development of resistant cell populations since drug-sensitive cells will survive during selection.

Cancer cells are known to be in a constant state of transition between proliferating and quiescent states [35]. The resulting heterogeneity will affect the sensitivity to cell cycle-active drugs such as DNA damaging agents and mitotic inhibitors and based on this study also affects sensitivity to b-AP15. Quiescent cells are known to show decreased levels of protein synthesis [53] and it is not unexpected that non-cycling cells will show decreased sensitivities of proteasome DUB inhibition. Consistent with this interpretation is the finding that quiescent multiple myeloma cells survive exposure to bortezomib [54]. In distinction to the findings reported here, quiescence was induced by bortezomib resulting in cell survival. The phenomenon of evasion from b-AP15-induced cell death is similar to the mechanism of relapse of tumors after treatment with platinum drugs. Slowly cycling cells present in the cell population were shown insensitive to platinum treatment and were able to reenter the cell cycle and repopulate tumors [55].

We were able to develop a cell line that showed limited resistance to b-AP15 and VLX1570. This cell line was found to be cross-resistant to doxorubicin and melphalan. Resistance was reversed by BSO treatment, an inhibitor of γ-glutamylcysteine synthetase. Resistance is associated with significantly increased GSH levels in this study, and HCT116^b-AP15^ cells maintain higher levels of GSH even at higher concentrations of b-AP15. A2780/BBR cells, displaying a tendency of decrease sensitivity to b-AP15 in comparison with their parental A2780 cells, show increased GSH transferase activity [50]. The sensitivity to 2cPE, a DUB inhibitor with a structure similar to that of b-AP15, was also reported to be augmented by BSO [56]. The dual enone structures of b-AP15/VLX1570 are expected to bind GSH, but we have not observed a major effect of GSH addition using *in vitro* assays [30] and we also did not observe an effect of BSO on the sensitivity of the parental HCT116 cells. These observations show a role of GSH conjugation in the development of resistance to b-AP15.

Analysis of cell lines overexpressing ABC transporters did not show any role of drug transporters in b-AP15 sensitivity. Drug uptake was not affected in b-AP15-selected HCT116 cells, consistent with this view.

We conclude from this study that resistance to b-AP15, in distinction to bortezomib, is difficult to develop *in vitro*. If this class of compounds can be developed into clinically useful therapeutic drugs, slow development of low levels of resistance will be beneficial to treatment outcomes.

## Experimental Procedures

### Reagents

b-AP15, VLX1570 and other analogues were synthesized by OnTarget Chemistry AB (Uppsala, Sweden). ^3^H-VLX1570 was synthesized by OnTarget Chemistry and had a specific activity of 2.0 MBq/mmol (radiochemical purity > 97%).

### Cell culture

The human colon carcinoma cell line HT29 (ATCC® HTB-38™, ATCC, Manassas VA, USA) and the mitoxantrone-resistant HT29/MIT subline [49] were maintained as monolayers in RPMI 1640 medium (Lonza, Basel, Switzerland), whereas LoVo (ATCC® CCL-229™, ATCC) and LoVo/DX cells [49] were maintained in F-12 medium (Lonza), both with 10% FBS (Gibco, Life Technologies, Carlsbad, California). The human ovarian carcinoma cell lines A2780 and the platinum-resistant variant A2780/BBR [50] were grown in RPMI-1640 plus 10% FBS. U2-OS and the derived stable transfectants with pCMV6-Neo vector or vector containing MRP1 and MRP4 were maintained in McCoy’s 5A medium plus 10% FBS and stable transfectants were kept in the presence of 400 mg/ml G418 [57]. All cell lines were maintained in a humidified atmosphere with 5% CO_2_ at 37°C.

HCT116 colon carcinoma cells (ATCC® CCL-247™, ATCC) were maintained in McCoy´s 5A modified medium/10% Fetal Bovine Serum (Gibco, Life Technologies). MelJuSo cells expressing yellow fluorescent protein (YFP) tagged ubiquitin (MelJuSo Ub^G76V^ YFP [36]) were cultured in Dulbecco’s Modified Eagle Medium (DMEM)/10% fetal calf serum (Gibco, Life Technologies). Cultures were maintained by growing cells to 75–80% confluence, followed by splitting cells 1:10. b-AP15- and bortezomib-resistant HCT116 cells were allowed to attach overnight after detachment, followed by continuous exposure to b-AP15 (250 nM) or bortezomib (100 nM). Resistant cells were actively cultured in this manner for ten months prior to experiments. Cells were routinely checked for mycoplasma contamination (Lonza), used within 20 passages from thawing from a frozen stock.

### Cell cycle analysis

Cell proliferation during drug treatment was visualized by live cell imaging using an Incucyte Zoom® instrument (Essen Bioscience, Ann Arbor MI, USA). MelJuSo Ub^G76V^ YFP cells were used as a reporter line for proteasome activity. Cells were treated with 250 nM b-AP15 and imaged every 10 minutes. Lineage tracing of cells was performed using these captures.

Flow cytometry was used to analyze cell cycle distribution. For determination of cell cycle HCT-116 cells were treated with b-AP15 (1μM), bortezomib (200nM), or DMSO as control. Cells were harvested by trypsinization then washed and fixed in 70% ice cold EtOH for 12 h. Cells were re-suspended in staining solution containing propidium iodide (50 μg/ml) and RNAse A (0.5 μg/ml) in PBS. Flow cytrometry was performed using the BD FACScalibur. Data was analyzed using ModFit software.

Protein levels of cyclins were analyzed using western blotting. HCT116 cells were treated with b-AP15 (0.5μM) for 6 hours. After gel electrophoresis proteins were detected with indicated antibodies at manufacturer’s recommended conditions (Cyclin Antibody Sampler Kit, Cell Signal Technology, Danvers MA, USA).

### *In vitro* selection for drug resistance

Resistance to b-AP15 was assessed by clonogenic assay. HCT116 cells were seeded at 90 cells/cm^2^ in 6 cm culture dishes. After attachment, cells were treated with 50 nM b-AP15 for four hours, followed by washout of the compound. Clones were allowed to grow for two weeks after washout. Surviving clones were reseeded and grown to 75–80% confluence in a culture flask, indicating recovery from treatment. To assess whether the surviving clones were resistant to b-AP15, the clonogenic assay was repeated on these cells. Clones surviving this second wave of treatment were again grown for two weeks after drug washout before fixation and staining in methanol blue.

Cell sensitivity to b-AP15 was further monitored using the MTT (3-[4,5-Dimethylthiazol-2-yl]-2,5-diphenyl-tetrazolium bromide) assay or cell counting. Cells were seeded into 96-well flat-bottom plates and exposed to a concentration range of b-AP15. After 48 h exposure, MTT was added to each well to a final concentration of 0.45 mg/ml. Plates were incubated at 37°C for four hours, culture medium was gently removed, and crystals were dissolved in DMSO on a shaker for 20 minutes. Absorbance was measured at 540 nm, with a reference reading at 650 nm.

For cell counting experiments, cells were seeded in 12-well plates and 24 h later exposed for 72 h to increasing concentrations of b-AP15. At the end of treatment, cell growth inhibition was evaluated by counting cells (Z2 Particle Counter, Beckman Coulter, Milan, Italy). All experiments were performed at least three times. IC_50_ is defined as the concentration of a drug inhibiting 50% of cell growth.

### Deubiquitinase activity

For deubiquitinase inhibition assays, cell lysates containing 10 μg protein were prepared from HCT116, b-AP15- and bortezomib-resistant cells in reaction buffer (50 mM HEPES, 250 mM sucrose, 5 mM MgCl2, 2 mM ATP, 1 mM DTT). Total DUB activity was analyzed using DUB substrate Ub-rhodamine 110 (0.5 μM). Absorbance was measured using a Spark^TM^ 10 M multimode microplate reader equipped with 485 nm excitation and 530 nm emission filters. For HA-UbVS labeling, cells were lysed in lysis buffer (50 mM HEPES, 250 mM sucrose, 5mM MgCl2, 50 mM NaCl, 0.5% Triton x-100, 2 mM ATP, 1 mM DTT) on ice for 20 min followed by removal of debris by centrifugation. 25μg protein was labeled with 1 μM HA-Ub-VS for 30 min at 37°C and then subjected to western blotting.

### 20S proteasome activity

Cell lysates containing 10μg protein were prepared from HCT116, b-AP15- and bortezomib-resistant cells in reaction buffer (25 mM HEPES, 0.5 mM EDTA and 0.03% sodium dodecyl sulfate). Chymotryptic-like activity of the 20S proteasome was analyzed using 20S substrate Suc-LLVY-AMC. Absorbance was measured using a Spark^TM^ 10M multimode microplate reader equipped with 351 nm excitation and 430 nm emission filters.

### Glutathione analysis

Depletion of gluthatione was achieved by addition of 10 μM BSO and 24 hours incubation of the cells in the culture flask prior to use of the cells. Non-treated cells were cultivated with similar conditions in parallel as a control. Fluorometric microculture cytotoxicity assay (FMCA) was performed according to the protocol by Lindhagen et al. [58]. In short, 180 μL cell suspension parental and b-AP15-resistant HCT116 cells, with or without BSO pretreatment, and 20 μL drug solution were added into the wells of a 96-well Nunc microtiter plate (Thermo Scientific, Waltham, MA, USA). Samples were incubated for 72 hours (37°C, humidified atmosphere, 5% CO2). Cells were washed once with PBS, and fluorescein diacetate (FDA; Sigma-Aldrich) was added (200 μL, 10 μg/mL) and plates were incubated for another 40 minutes before measurement of fluorescence (485/538 nm) in a FLUOstar Omega (BMG Labtech, Ortenberg, Germany). Each individual experiment was performed at least three times. From the mean SI%-logConcentration curves the half maximal inhibitory concentration (IC_50_) was determined using non-linear regression analysis in Prism 5 Software Package (Graph Pad, La Jolla, CA, USA).

For assessment of GSH levels under treatment with b-AP15, HCT116^wt^ and HCT116^b-AP15^ cells were seeded at a concentration of 5000 cells/well in opaque 96 well culture plates (Nunc, Rokskilde, Denmark). Cells were treated with the indicated concentration range of b-AP15 for 6 hours, followed by immediate removal of culture medium. GSH levels were measured using GSH-Glo^tm^ kit (Promega, Madison WI, USA) according to manufacturer’s instructions. Briefly, cells were lysed for 30 minutes in buffer containing Luciferin-NT and Glutathione-S-Transferase (GST), at concentrations where GST effectively catalyzes the generation of Luciferin from Luciferin-NT proportional to the concentration of GSH in the sample. Luciferin was then detected as a luminescent signal by incubation in Luciferin Detection Reagent containing Luciferase for 15min. Luminescence was measured using the GloMax® Exporer Microplate Reader (Promega, Madison WI, USA).

### Drug uptake

Cells were incubated in the presence of [3H]-labelled VLX1570 for 60 minutes. Medium was discarded and cells washed 3 times in ice-cold PBS. Cells were collected by scraping and radioactivity was determined in a MicroBeta scintillation counter (PerkinElmer, Waltham MA, USA).

## Supporting information

S1 Fig Shown is the cell cycle time (from last mitosis) of individual cells at the time of drug addition on the x-axis. The time to cell death of each cell is plotted on the y-axis. Note the absence of correlation.
(EPS)

S1 Table Analysis of correlations between b-AP15 sensitivity and ABC drug transporter expression. Data on b-AP15 sensitivity was retrieved from the dtp.cancer.gov website and ABC drug transporter expression from reference [43]. Shown are selected transporters from the 48 reported. A negative correlation is expected if a specific reporter indeed affects sensitivity. Both positive and negative correlations were observed. None of these significances were significant after adjustment for multiple correlations (p < 0,001).
(DOCX)

S2 Table Analysis of correlations between b-AP15 sensitivity and solute carrier (SLC) family expression. Data on b-AP15 sensitivity was retrieved from the dtp.cancer.gov website and solute carrier expression from reference [44]. Both positive and negative correlations were observed, none being statistically significant at p = 0.05.
(DOCX)
