# Supplementary material for: Analysis of determinants for in vitro resistance to the small molecule deubiquitinase inhibitor b-AP15
Source: PLoS One. 2019 Oct 22;14(10):e0223807. doi: 10.1371/journal.pone.0223807 (PMC6804958; doi:10.1371/journal.pone.0223807)
Supplement: S1 Table — Data on b-AP15 sensitivity was retrieved from the dtp.cancer.gov website and ABC drug transporter expression from reference [43]. Shown are selected transporters from the 48 reported. A negative correlation is expected if a specific reporter indeed affects sensitivity. Both positive and negative correlations were observed. None of these significances were significant after adjustment for multiple correlations (p < 0,001). (DOCX) [file pone.0223807.s002.docx]

**S1 Table. Analysis of correlations between b-AP15 sensitivity and ABC drug transporter expression.**

| **ABC efflux pump** | **Spearman R** | **P-value** |
| --- | --- | --- |
| ABCB1 (MDR1) | 0.0068 | NS |
| ABCC1 (MRP1) | 0.3 | p = 0.027 |
| ABCC4 (MRP4) | 0.17 | p = 0.22 |
| ABCG2 (BCRP) | 0.14 | p = 0.10 |
| ABCA13 | 0.38 | p = 0.004 |
| ABCB2 | -0.36 | p = 0.006 |
| ABCB7 | -0.34 | p = 0.009 |
| ABCD1 | 0.32 | p = 0.016 |

Data on b-AP15 sensitivity was retrieved from the dtp.cancer.gov website and ABC drug transporter expression from reference [44]. Shown are selected transporters from the 48 reported. A negative correlation is expected if a specific reporter indeed affects sensitivity. Both positive and negative correlations were observed. None of these significances were significant after adjustment for multiple correlations (p < 0,001).
