# Supplementary material for: Analysis of determinants for in vitro resistance to the small molecule deubiquitinase inhibitor b-AP15
Source: PLoS One. 2019 Oct 22;14(10):e0223807. doi: 10.1371/journal.pone.0223807 (PMC6804958; doi:10.1371/journal.pone.0223807)
Supplement: S2 Table — Data on b-AP15 sensitivity was retrieved from the dtp.cancer.gov website and solute carrier expression from reference [44]. Both positive and negative correlations were observed, none being statistically significant at p = 0.05. (DOCX) [file pone.0223807.s003.docx]

**S2 Table. Analysis of correlations between b-AP15 sensitivity and solute carrier (SLC) family expression.**

| **Transporter** | **Spearman R** |
| --- | --- |
| SLCO1A2 | 0.10 |
| SLCO1B1 | 0.20 |
| SLCO1B3 | -0.14 |
| SLCO1C1 | 0.14 |
| SLCO2A1 | -0.001 |
| SLCO2B1 | 0.083 |
| SLCO3A1 | 0.083 |
| SLCO4A1 | -0.14 |
| SLCO4C1 | 0.06 |
| SLCO5A1 | 0.013 |
| SLCO6A1 | -0.19 |
| SLC22A1 | -0.19 |
| SLC22A2 | -0.67 |
| SLC22A3 | 0.081 |
| SLC22A4 | 0.008 |
| SLC22A5 | 0.21 |
| SLC22A11 | -0.13 |
| SLC22A13 | -0.15 |
| SLC22A14 | 0.089 |
| SLC22A15 | 0.14 |
| SLC22A16 | 0.13 |
| SLC22A17 | 0.18 |
| SLC22A18 | 0.22 |

Data on b-AP15 sensitivity was retrieved from the dtp.cancer.gov website and solute carrier expression from reference [44]. Both positive and negative correlations were observed, none being statistically significant at p = 0.05.
